# Highly Pathogenic Avian Influenza A(H5N1) in Wild Birds and a Human, British Columbia, Canada, 2024

**DOI:** 10.3201/eid3106.241862

**Published:** 2025-06

**Authors:** Chelsea G. Himsworth, Jessica M. Caleta, Agatha N. Jassem, Kevin C. Yang, James E.A. Zlosnik, John R. Tyson, Laurie Wilson, Kevin S. Kuchinski, Jolene Giacinti, Megan Willie, Tony D. Redford, Maeve Winchester, Caeley Thacker, Yohannes Berhane, Theresa Burns, Natalie Prystajecky, Shannon L. Russell

**Affiliations:** British Columbia Ministry of Agriculture and Food, Abbotsford, British Columbia, Canada (C.G. Himsworth, T.D. Redford, T. Burns); British Columbia Centre for Disease Control, Vancouver, British Columbia, Canada (J.M. Caleta, A.N. Jassem, K.C. Yang, J.E.A. Zlosnik, J.R. Tyson, K.S. Kuchinski, N. Prystajecky, S.L. Russell); Canadian Wildlife Service–Environment and Climate Change Canada, Delta, British Columbia, Canada (L. Wilson, M. Willie); Environment and Climate Change Canada, Ottawa, Ontario, Canada (J. Giacinti); British Columbia Ministry of Water, Land and Resource Stewardship, Nanaimo, British Columbia, Canada (M. Winchester, C. Thacker); Canadian Food Inspection Agency, Winnipeg, Manitoba, Canada (Y. Berhane)

**Keywords:** Highly pathogenic avian influenza A, wild birds, influenza A virus, HPAI, H5N1, influenza, zoonoses, respiratory infections, British Columbia, Canada

## Abstract

We characterized highly pathogenic avian influenza A(H5N1) clade 2.3.4.4b genotype D1.1 in wild birds and a human in British Columbia, Canada, during 2024. D1.1, the predominant genotype circulating in fall 2024, is a reassortment between Eurasian A3 lineage viruses, introduced to North America in 2022, and North American lineage viruses.

In fall 2021, highly pathogenic avian influenza (HPAI) A(H5N1) virus clade 2.3.4.4b was introduced into wild birds and domestic poultry in eastern Canada via the East Atlantic Flyway (*1*). It subsequently spread throughout North America before arriving in British Columbia, Canada, in April 2022 (*1*). A second incursion of HPAI H5N1 virus, clade 2.3.4.4b, brought in by the Pacific Flyway (genotype A3) (*2*), occurred in February 2022, resulting in both viruses circulating among wild birds in the province and causing numerous spillover events into poultry (*3*). The virus affected more poultry flocks in British Columbia than in any other province in Canada (*4*), likely because high-density poultry farming is co-located with optimal habitat for overwintering waterfowl in the Fraser Valley (*3*).

As of the end of 2024, British Columbia had endured 4 distinct waves of HPAI H5N1 clade 2.3.4.4b virus; each wave was characterized by increased detections in wildlife and poultry, the emergence of new genotypes (Table), and shifts in the dominant genotype (Figure 1). Most circulating HPAI H5N1 clade 2.3.4.4b viruses in the first 3 waves were reassortant descendants of the virus introduced via the East Atlantic Flyway (*3*); however, in fall 2024, a new wave of infections occurred in British Columbia wild bird populations associated primarily with a novel genotype (D1.1, 3) that was a descendant of the A3 genotype. We used whole-genome sequencing and phylogenetic analysis of HPAI H5N1 clade 2.3.4.4b viruses detected in wild birds in British Columbia during October and November 2024 to describe the features, ecology, and possible origins of this genotype.

**Table:** Reassortant classification and genotypic characterization of clade 2.3.4.4b viruses in a study of highly pathogenic avian influenza A(H5N1) in wild birds and a human, British Columbia, Canada, 2024*

GenoFLU genotype†	Genotype assignment by influenza A segment*								Outbreak wave‡
	HA	NA	M	NP	NS	PA	PB1	PB2	
B2.1	EA1	EA1	EA1	Am1.1	EA1	EA1	EA1	Am1.2	Wave 1
B3.2	EA1	EA1	EA1	Am1.4.1	Am1.1	EA1	Am1.2	Am2.1	Waves 1–3
B3.6	EA1	EA1	EA1	Am1.4.1	Am1.1	EA1	Am4	Am5	Wave 3
B3.10	EA1	EA1	EA1	Am4	Am1.1	EA1	Am4	Am5	Wave 3
B3.1	EA1	EA1	EA1	Am1.4.1	EA1	EA1	EA1	Am2.1	Wave 1
B4.1	EA1	EA1	EA1	Am1.3	EA1	EA1	EA1	Am2.2	Waves 1–3
A3	EA3	EA3	EA3	EA3	EA3	EA3	EA3	EA3	Waves 1–4
D1.1	EA3	Am4N1	EA3	Am13	EA3	Am4	EA3	Am24	Wave 4
B3.13	EA1	EA1	EA1	Am8	Am1.1	EA1	Am4	Am2.2	US cattle genotype (for context only)

*Data show viruses detected in British Columbia, Canada, during September 2021–November 2024 contextualized by the US cattle virus genotype B3.13 identified in 2024. Am, North American lineage; EA, Eurasian lineage; HA, hemagglutinin; M, matrix; NA, neuaminidase; NP, nucleoprotein; NS, nonstructural; PA, polymerase acidic; PB, polymerase basic. †Genotype assignment from GenoFlu pipeline (*2*). ‡Wave 1, April–Sept 2022; wave 2, Sept 2022–Aug 2023; wave 3, Sept 2023–Aug 2024; wave 4, Sept 2024–November 2024.

**Figure 1:**
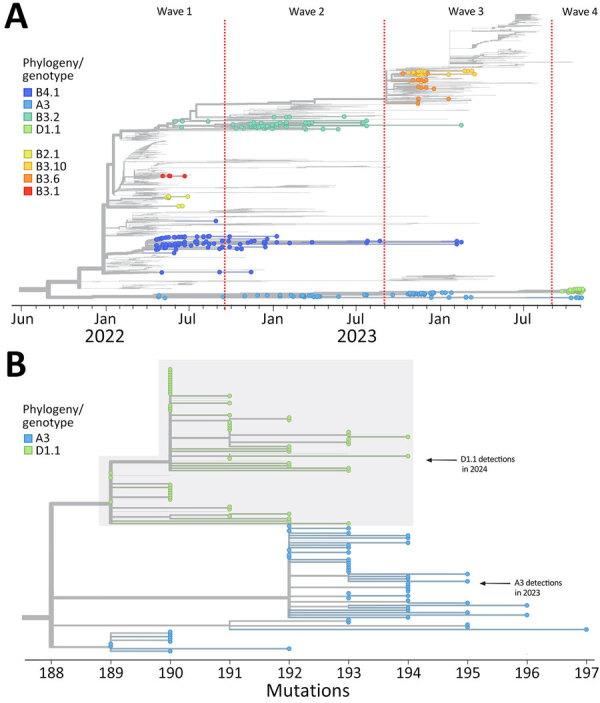
Hemagglutinin-specific phylogenetic analysis of clade 2.3.4.4b detections from a study of highly pathogenic avian influenza A(H5N1) in wild birds and a human, British Columbia, Canada, 2024. Data based on detections during September 2021–November 2024 contextualized by sequences from other parts of North and South America. Trees are rooted on the A/goose/Guangdong/1/96 (H5) reference sequence. A) Detections of all genotypes plotted on the basis of specimen collection date. B) Genotype A3 and D1.1 detections plotted on the basis of divergence.

## The Study

As part of initiatives driven by the British Columbia Wildlife Avian Influenza Surveillance Program, we screened oropharyngeal and cloacal swab specimens collected from wild bird carcasses using quantitative reverse transcription PCR targeting a conserved region in the matrix gene and subjected positive samples (cycle threshold <36) to whole-genome sequencing (*3*). Sequences underwent subtyping, genotyping, and phylogenetic analysis as previously described (*3*).

During October 3, 2024–November 8, 2024, there were 57 detections of HPAI H5N1clade 2.3.4.4b virus in wild birds in British Columbia, including 6 detections of genotype A3 and 51 detections of genotype D1.1 (Figure 1, panel A). Of note, A3 viruses identified in BC in 2024 were more closely related to A3 viruses from Japan (2024) than A3 viruses from British Columbia in 2023, suggesting that they represent a new HPAI incursion from East Asia (Appendix Figure). Most (68.6%, 35/51) detections occurred in the Fraser Valley. Across the province, detections occurred predominantly in cackling geese (*Branta hutchinsii*; 35.3%, 18/51), followed by Canada geese (*Branta canadensis*; 17.6%, 9/51) and snow geese (*Anser caerulenscens*; 15.7%, 8/51); 1–3 detections each occurred in American wigeons (*Mareca americana*), bald eagles (*Haliaeetus leucocephalus*), barred owls (*Strix varia*), great blue herons (*Ardea herodias*), great horned owls (*Bubo virginianus*), green-winged teals (*Anas carolinensis*), northern pintails (*Anas acuta*), peregrine falcons (*Falco peregrinus*), and red-tailed hawks (*Buteo jamaicensis*).

**Figure 2:**
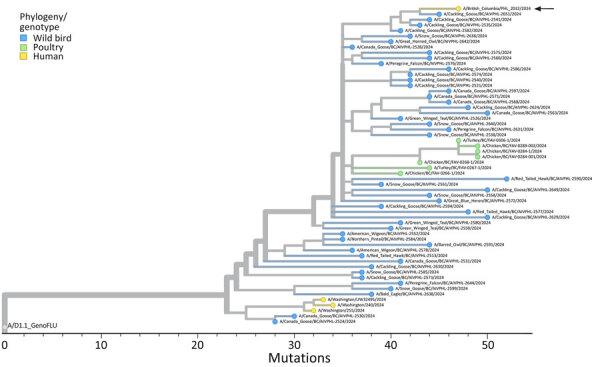
Phylogenetic analysis of concatenated full genome sequences of clade 2.3.4.4b genotype D1.1 detections in wild birds, poultry, and a human from a study of highly pathogenic avian influenza A(H5N1) in wild birds and a human (arrow), British Columbia, Canada, 2024. Data drawn from detections during October 3–November 8, 2024. Tree is plotted based on divergence and rooted to a composite D1.1 reference sequence assembled from D1.1 segments obtained from the GenoFLU Version 1.0.5 database (https://github.com/USDA-VS/GenoFLU/).

On November 8, 2024, we confirmed a diagnosis of influenza caused by HPAI H5N1 clade 2.3.4.4b genotype D1.1 in a teenager from the Fraser Valley (*5**)*. The sequence we obtained from this patient was most closely related to viruses detected in wild birds (Figure 2). The D1.1 lineage strain of the virus has also been associated with all but one of the 60 poultry outbreaks that occurred in British Columbia during October 21–November 30, 2024 (*4*).

**Figure 3:**
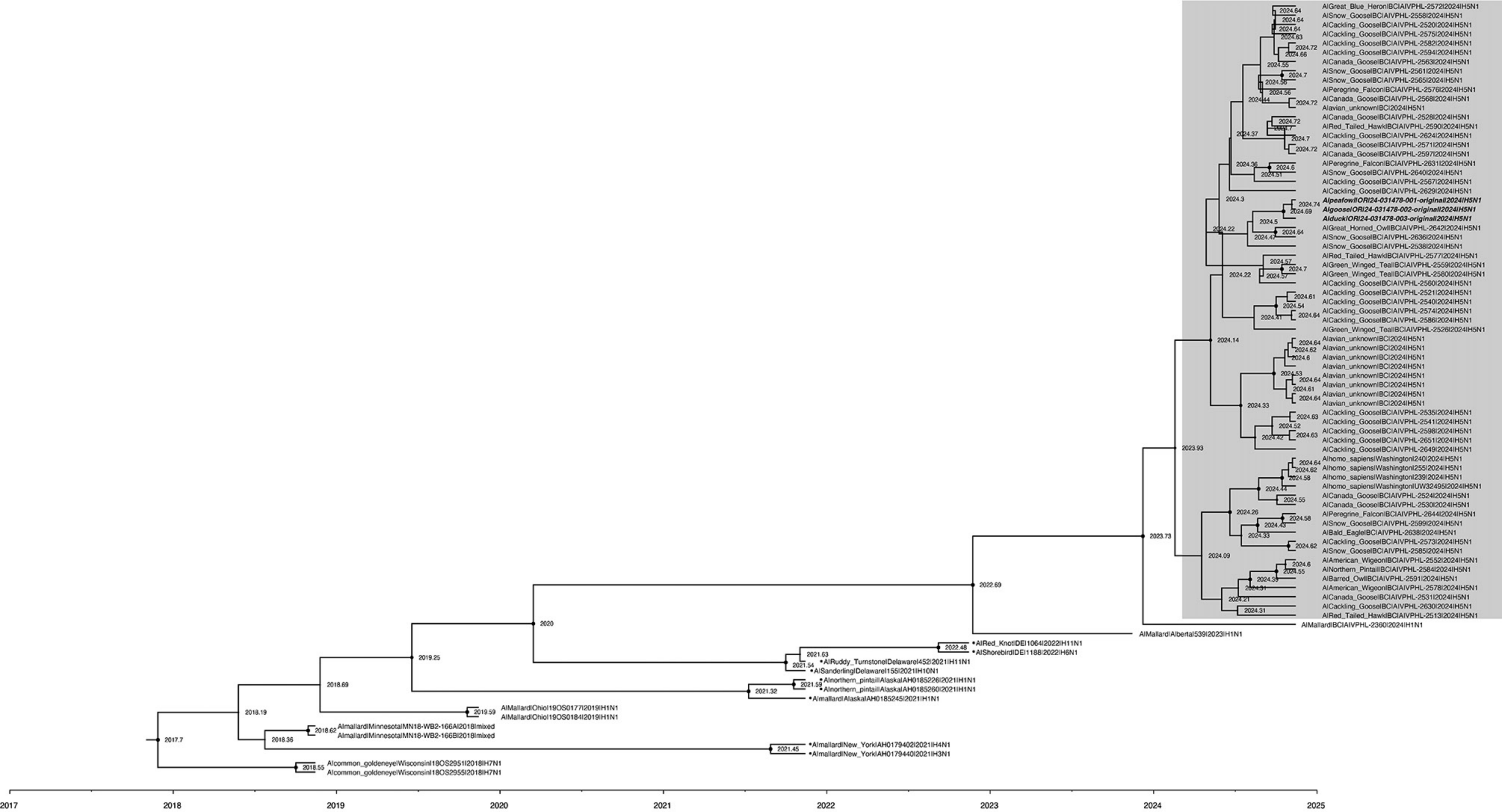
Phylogenetic relationships and possible origins of the neuraminidase (NA) segment (Am4N1) of the clade 2.3.4.4b D1.1 genotype detected in fall 2024 as part of a study of highly pathogenic avian influenza A(H5N1) in wild birds and a human, British Columbia, Canada, 2024. The maximum clade credibility tree was inferred by using BEAST v2.7.7 (*6*), incorporating NA gene sequences from D1.1 viruses and closely related sequences from wild birds detected in British Columbia and National Center for Biotechnology Information databases. Analysis used an uncorrelated relaxed log-normal clock, the Hasegawa-Kishino-Yano substitution model without gamma rate heterogeneity, and a coalescent Bayesian skyline tree prior. The posterior distribution was approximated using 100 million Markov chain Monte Carlo steps, sampled every 10,000 steps, with a 10% burn-in. All NA segments were identified as Am4N1 by GenoFLU except those indicated with a black dot on the tree tip label; non-Am4N1 sequences were classified as unassigned. Shaded box indicates NA segments from D1.1 and D1.2 H5N1 viruses. D1.2 samples are bolded and italicized. Node support values and branch lengths indicate the evolutionary divergence and probable reassortment timeframes leading to the emergence of the Am4N1 NA segment in the D1.1 genotype.

The D1.1 genotype contained 4 Eurasian lineage segments (hemagglutinin, matrix, nonstructural, and polymerase basic 1) that were related to the 2023 BC A3 lineage viruses and 4 segments from North American lineage viruses (neuraminidase [NA], nucleoprotein, polymerase acidic, and polymerase basic 2), including an NA segment not previously associated with HPAI H5N1 clade 2.3.4.4b viruses in British Columbia (Table). Although the 2024 D1.1 lineage viruses and the 2023 A3 lineage viruses shared a recent common ancestor, the 2024 D1.1 lineage viruses displayed less genetic divergence than expected over that intervening time (Figure 1, panel B). Phylodynamic analysis using BEAST (https://beast.community) and sequences from National Center for Biotechnology Information and GISAID (https://www.gisaid.org) databases revealed that the closest relative to the D1.1 Am4N1 NA segment was an H1N1 virus detected in a mallard (A/mallard/BC/AIV-PHL-2360/2024) from the British Columbia interior on August 20, 2024, and that the reassortment event involving that segment may have occurred in fall 2023 (Figure 3) (*6*). That H1N1 virus belongs to a lineage of earlier H1N1 viruses also detected in mallards and was distantly related to another H1N1 detected in Alberta (A/Mallard/AB/539/2023). Of note, several Am4N1 segments from D1.1 viruses in poultry from the same British Columbia outbreak (A/chicken/BC/FAV-0289-002/2024, A/chicken/BC/FAV-0284-001/2024, A/turkey/BC/FAV-0306-1/2024) had a mutation associated with antiviral resistance, NA-H275Y (*7*); however, that mutation was not detected in any of the wild bird sequences.

## Conclusions

The once dominant B genotypes associated with the HPAI H5N1 clade 2.3.4.4b virus incursion via the Eastern Atlantic Flyway do not appear to be circulating in wild birds within the Pacific Flyway as of fall 2024 (despite the continuing presence of genotype B3.13 in cattle in the western United States [*8*]) (Table). Instead, a novel genotype, D1.1, has emerged that is the result of a reassortment among the A3 genotypes originally introduced via the Pacific Flyway and >1 North American lineage avian influenza viruses. This virus spilled over into poultry in British Columbia and infected 1 human and has also been detected in parts of the United States south of British Columbia (Figure 2).

Compared with A3 viruses detected a year earlier, the hemagglutinin segment of the D1.1 viruses had acquired fewer net substitutions than expected, despite sharing a relatively recent common ancestor (Figure 1, panel B). This finding could suggest that the D1.1 genotype or its ancestors may have been preserved in an environmental reservoir—e.g., in frozen wetlands in the high arctic (*9*)—in the summer of 2024 before being reintroduced into migratory birds in the fall. Alternatively, this viral lineage may be particularly well adapted to certain wild bird species or populations, resulting in circulation with minimal evolutionary pressure. This finding has implications for the use of molecular clock theory in phylodynamic modeling of HPAI viruses.

We note that D1.1 appears to be unique among the HPAI H5N1 clade 2.3.4.4b genotypes due to the acquisition of a North American lineage NA segment. The Am4N1 NA segment likely originated from a reassortment event involving waterfowl in western Canada, potentially within British Columbia. Further studies are needed to determine where and when the other reassorted segments were acquired.

Of interest, the prevalence of environmental HPAI H5N1 virus clade 2.3.4.4b detections based on genomic analysis of wetland sediment was far greater in fall 2024 than for data from fall 2023 (*10*). This phenomenon could suggest that a great number of birds were infected with D1.1 compared with other genotypes in previous years, that the genotype is associated with greater viral shedding, or both. The NA segment encodes the enzyme required for viral release from infected cells (*11*). Certain NA lineages, therefore, might increase viral shedding in wild birds. Considering the explosive wave of poultry outbreaks observed in British Columbia in late 2024, it would be prudent to investigate whether the Am4N1 NA segment has a functional impact on host-virus interactions. In addition, it will be important to determine the implications of D1.1 for host range and infectivity, given that the virus detected in the human case was most closely related to those found in wild birds, suggesting the potential for direct or indirect transmission from wild birds to humans.

Appendix Additional information for highly pathogenic avian influenza A in wild birds and a human, British Columbia, Canada, 2024.
