# Supplementary material for: Highly Pathogenic Avian Influenza A(H5N1) in Wild Birds and a Human, British Columbia, Canada, 2024
Source: Emerg Infect Dis. 2025 Jun;31(6):1216–21. doi: 10.3201/eid3106.241862 (PMC12123910; doi:10.3201/eid3106.241862)
Supplement: Appendix — Additional information for highly pathogenic avian influenza A in wild birds and a human, British Columbia, Canada, 2024. [file 24-1862-Techapp-s1.pdf]

Article DOI: http://doi.org/10.3201/eid3106.241862

EID cannot ensure accessibility for supplementary materials supplied by authors. Readers who have difficulty accessing supplementary content should contact the authors for assistance.

Highly Pathogenic Avian Influenza A in Wild Birds and a Human, British Columbia, Canada, 2024

Appendix

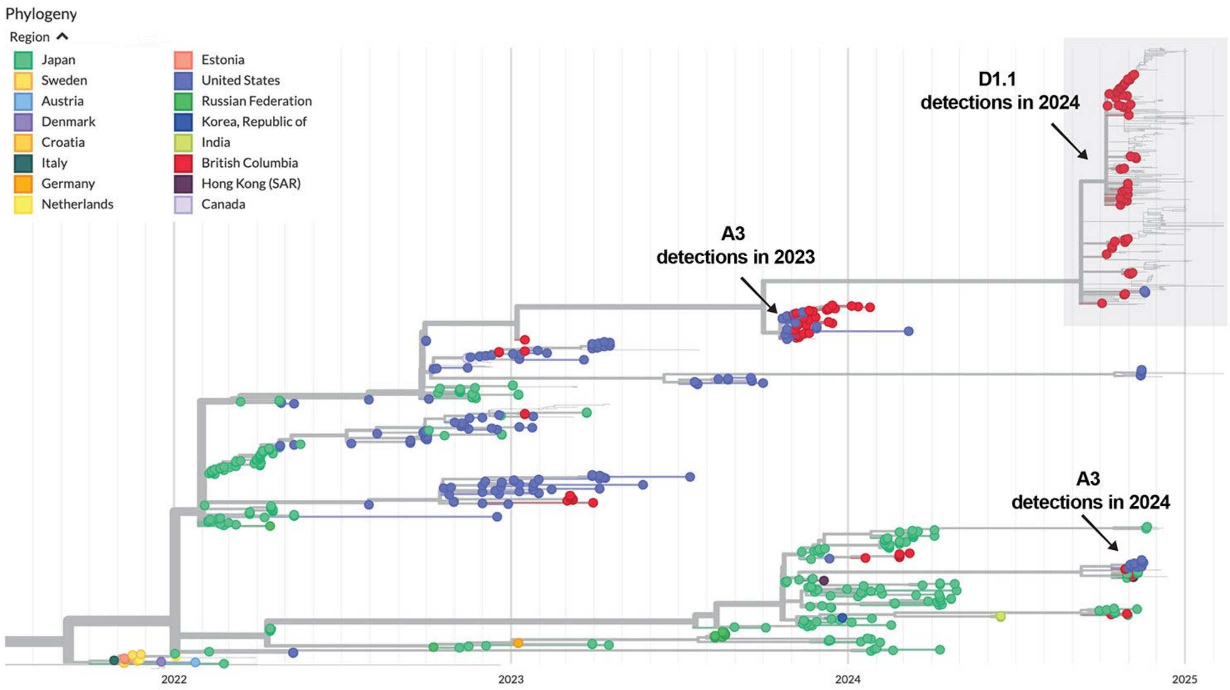

Appendix Figure. Hemagglutinin (HA)-specific phylogenetic analysis of HPAI A(H5N1) virus, clade 2.3.4.4b detections in wild birds and a human from British Columbia, Canada between September 2021 and November 2024 contextualized by global sequences. Trees are rooted on the A/Goose/Guangdong/1/96 (Gs/Gd) (H5) reference sequence. Phylogeny displayed represent EA3 genotype HA segments, represented by A3 and D1.1 viruses. Detections plotted based on specimen collection date. Grey shaded box indicates D1.1 viruses, all other sequences shown are A3 viruses. Tree tip color indicates collection region identified in GISAID metadata. Grey hashed lines represent additional sequences pulled from GISAID (submitted Dec 1, 2024–Feb 18, 2025), but not included in the original D1.1 analysis.
